# Supplementary material for: Mid-infrared analogue polaritonic reversed Cherenkov radiation in natural anisotropic crystals
Source: Nat Commun. 2023 May 3;14:2532. doi: 10.1038/s41467-023-37923-w (PMC10156754; doi:10.1038/s41467-023-37923-w)
Supplement: Supplementary file 1 — supplementary information [file 41467_2023_37923_MOESM1_ESM.pdf]

Supplementary Information for

"Mid-infrared analogue polaritonic reversed Cherenkov radiation in natural anisotropic crystals"

Xiangdong Guo[1,2#], Chenchen Wu[1,2#], Shu Zhang[1,2], Debo Hu[1,2], Shunping Zhang[3], Qiao Jiang[4], Xiaokang Dai[1], Yu Duan[1], Xiaoxia Yang[1,2], Zhipei Sun[5], Shuang Zhang[6], Hongxing Xu[3], and Qing Dai[1,2]**

[1] CAS Key Laboratory of Nanophotonic Materials and Devices, CAS Key Laboratory of Standardization and Measurement for Nanotechnology, CAS Center for Excellence in Nanoscience, National Center for Nanoscience and Technology, Beijing 100190, China

[2] Center of Materials Science and Optoelectronics Engineering, University of Chinese Academy of Sciences, Beijing 100049, China

[3] School of Physics and Technology, Center for Nanoscience and Nanotechnology, and Key Laboratory of Artificial Micro- and Nano-structures of Ministry of Education, Wuhan University, Wuhan 430072, China

[4] College of Physics, Chongqing University, Chongqing 401331, China.

[5] Department of Electronics and Nanoengineering and QTF Centre of Excellence, Department of Applied Physics, Aalto University, Espoo 02150, Finland.

[6] Department of Physics, University of Hong Kong, Hong Kong 999077, China.

[#]These authors contributed equally: Xiangdong Guo, Chenchen Wu.

E-mail: daiq@nanoctr.cn, yangxx@nanoctr.cn

This PDF file includes:

Supplementary Notes 1-3

Supplementary Figures S1-S11

Supplementary References

Supplementary Note 1: Simulation of fast-electrons excited phonon polaritons

When the flying electrons pass over the surface of MoO_3 material, the Cherenkov radiation (CR) of phonon polariton can be generated. The electron can be treated as a classical point source of charge -*e*, which can generate an external current density given by[1,2]

$$\mathbf{j}(x, y, z, \omega) = -e \ \hat{\boldsymbol{x}} \ \delta[\rho] e^{i\omega x/v} \tag{S1}$$

where the electron is taken to move with constant velocity vector v oriented along *x* axis, ρ represents the radiation plane. The velocity v= 0.2c is shown in Fig. 1c. The MoO_3 is modeled by its dielectric function, which is from the Lorentz model.[3,4] The electric field (E_z) distributions of polaritonic reversed CR above a MoO_3 layer at ω= 977 cm^{-1} are shown in Fig. 1c, after the electron passed through the simulation region. It can be seen that the electric field distribution exhibits polaritonic reversed CR, where the radiation cone is reversed to the trajectory of the moving electrons.

Supplementary Note 2: Dispersion and isofrequency contour (IFC) of MoO_3 phonon polaritons

To quantify the dispersion of MoO_3 phonon polaritons, the three-layer system (air/MoO_3/substrate) can be considered. We use the analytical dispersion of polariton propagation in a biaxial slab embedded between two semi-infinite media:[3-5]

$$q = \frac{\varphi}{k_0 d}\left[\arctan\left(\frac{\varepsilon_1 \varphi}{\varepsilon_z}\right) + \arctan\left(\frac{\varepsilon_3 \varphi}{\varepsilon_z}\right) + \pi l\right], l = 0,1,2 \ldots \tag{S2}$$

where the ε_1, and ε_3 correspond to air, and substrate, respectively, *d* is the thickness of the MoO_3 material, and k_0 is the wavevector of free space. $q = k_t/k_0$ is the normalized in-plane wavevector, where $k_t^2 = k_x^2 + k_y^2$.

$$\varphi = i\sqrt{\varepsilon_z/(\varepsilon_x cos^2\phi + \varepsilon_y sin^2\phi)}, \tag{S3}$$

where the $\varepsilon_{x,}$ $\varepsilon_{y,}$ and ε_z correspond to *x, y*, and *z* components of dielectric function in MoO_3 layer. The ϕ is the angle between the *x*-axis and the in-plane component vector. The equation (S2) not only shows the dispersion of the phonon polariton but also can calculate the in-plane IFCs. In addition, a false-color map of the phonon

polariton dispersion can also be calculated by the transfer-matrix[3], which is consistent with the analytical formula (S2).

Supplementary Note 3: The IFC of hBN/MoO_3 phonon polaritons

The hBN/MoO_3 can be treated as a laterally infinitely layered medium consisting of four layers (Supplementary Fig. S11): $z > 0$ (air, layer $j = 1$), $-d_1 < z < 0$ (hBN, layer $j = 2$), $-(d_1 + d_2) < z < -d_1$ (MoO_3, layer $j = 3$), and $z < -(d_1 + d_2) = -d$ (SiO_2, layer $j = 4$). Without loss of generality, we assume polaritons in hBN/MoO_3 propagate along the $k_{in-plane}$ direction in the xy plane. In the xyz coordinates, we have

$$k_x = k_{in-plane} \cdot \sin(\phi),\ k_y = k_{in-plane} \cdot \cos(\phi), \tag{S4}$$

where ϕ represents the angle between $\vec{k}_{in-plane}$ and the x axis. Thus the dielectric function of MoO_3 along the direction of $\vec{k}_{in-plane}$ can be expressed as $\varepsilon_{mt} = \varepsilon_{mx}\cos^2(\phi) + \varepsilon_{my}\sin^2(\phi)$.

Polaritons in MoO_3 are generally hybrid transverse magnetic-transverse electrical (TM-TE) eigenmodes, but the intensity of the TE mode is much weaker than the TM mode (TM: E_x, H_y, E_z)[6,7]. The transverse magnetic field in the hBN/MoO_3 heterostructure can be expressed as $\vec{H}_y = \vec{e}_y H_y(z) e^{(iqx - i\omega t)}$, which can satisfy the wave equation $\nabla^2 \vec{H} + \overleftrightarrow{\varepsilon} k_0^2 \vec{H} = \nabla(\nabla \cdot \vec{H})$. Thus

$$\frac{\partial^2 \vec{H}_y}{\partial z^2} + \left(\varepsilon_{a,s}^{(j)} k_0^2 - q^2\right) \vec{H}_y = 0, \tag{S5}$$

$$\frac{\partial^2 \vec{H}_y}{\partial z^2} + \left(\varepsilon_t^{(j)} k_0^2 - \frac{\varepsilon_t^{(j)}}{\varepsilon_z^{(j)}} q^2\right) \vec{H}_y = 0, \tag{S6}$$

There are evanescent fields in both the air layer and the substrate layer, so the interface can satisfy $\varepsilon_{a,s}^{(j)} k_0^2 - q^2 < 0,\ j = 0, 3$ and $\varepsilon_t^{(j)} k_0^2 - (\varepsilon_t^{(j)}/\varepsilon_z^{(j)}) q^2 > 0,\ j = 1, 2$. In the four-layer structure, the distribution of H_y satisfies:

$$H_y(z) = \begin{cases} (P_1 + P_2) e^{-Q_a z}, & z > 0 \\ P_1 e^{ik_z^{(1)} z} + P_2 e^{-ik_z^{(1)} z}, & -d_1 < z \le 0 \\ P_3 e^{ik_z^{(2)} z} + P_4 e^{-ik_z^{(2)} z}, & -d < z \le -d_1 \\ (P_3 e^{-ik_z^{(2)} d} + P_4 e^{ik_z^{(2)} d}) e^{-Q_s (z+d)}, & z \le -d \end{cases} \tag{S7}$$

According to $\nabla \times H = i\omega\varepsilon_0 \overleftrightarrow{\varepsilon} E$, Thus

$$E_x(z) = \begin{cases} \dfrac{iQ_a}{\omega\varepsilon_0\varepsilon_a}(P_1 + P_2)e^{-Q_a z}, & z > 0 \\ \dfrac{1}{\omega\varepsilon_0\varepsilon_t^{(1)}}(P_1 e^{ik_z^{(1)}z} + P_2 e^{-ik_z^{(1)}z}), & -d_1 < z \le 0 \\ \dfrac{1}{\omega\varepsilon_0\varepsilon_t^{(2)}}(P_3 e^{ik_z^{(2)}z} + P_4 e^{-ik_z^{(2)}z}), & -d < z \le -d_1 \\ \dfrac{-iQ_s}{\omega\varepsilon_0\varepsilon_s}(P_3 e^{-ik_z^{(2)}d} + P_4 e^{ik_z^{(2)}d})e^{-Q_s(z+d)}, & z \le -d \end{cases} \tag{S8}$$

Where $Q_{a,s} = \sqrt{-\varepsilon_{a,s}k_0^2 + q^2}$, and $k_z^{(1,2)} = \sqrt{\varepsilon_t^{(1,2)}k_0^2 - (\varepsilon_t^{(1,2)}/\varepsilon_z^{(1,2)})q^2}$.

Considering that the tangential components of $\vec{E}$ and $\vec{H}$ are continuous at the interface,

$$\begin{cases} E_x^{(0)} = E_x^{(1)}, & H_y^{(0)} = H_y^{(1)}, & z = 0 \\ E_x^{(1)} = E_x^{(2)}, & H_y^{(2)} = H_y^{(3)}, & z = -d_1 \\ E_x^{(2)} = E_x^{(3)}, & H_y^{(2)} = H_y^{(3)}, & z = -d \end{cases} \tag{S9}$$

Thus the coefficients (P_1-P_4) can satisfy the $M \cdot (P_1, P_2, P_3, P_4)^T = 0$,

$$M = \begin{pmatrix} \dfrac{iQ_a}{\varepsilon_a} - \dfrac{k_z^{(1)}}{\varepsilon_t^{(1)}} & \dfrac{iQ_a}{\varepsilon_a} + \dfrac{k_z^{(1)}}{\varepsilon_t^{(1)}} & 0 & 0 \\ \dfrac{k_z^{(1)}}{\varepsilon_t^{(1)}} e^{-ik_z^{(1)}d1} & -\dfrac{k_z^{(1)}}{\varepsilon_t^{(1)}} e^{ik_z^{(1)}d_1} & -\dfrac{k_z^{(2)}}{\varepsilon_t^{(2)}} e^{-ik_z^{(2)}d_1} & \dfrac{k_z^{(2)}}{\varepsilon_t^{(2)}} e^{ik_z^{(2)}d_1} \\ e^{-ik_z^{(1)}d1} & e^{ik_z^{(1)}d_1} & -e^{-ik_z^{(2)}d_1} & e^{ik_z^{(2)}d_1} \\ 0 & 0 & \left(\dfrac{iQ_s}{\varepsilon_s} + \dfrac{k_z^{(2)}}{\varepsilon_t^{(2)}}\right) e^{-ik_z^{(2)}d} & \left(\dfrac{iQ_s}{\varepsilon_s} - \dfrac{k_z^{(2)}}{\varepsilon_t^{(2)}}\right) e^{ik_z^{(2)}d} \end{pmatrix}$$

When the determinant det{M} = 0, it is guaranteed that there are non-zero solutions for the amplitude coefficients (P_1-P_4). Specifically, the dispersion relation can be stated as,

$$e^{2n_1} = -\frac{[(n_2-n_3)(n_3-n_4)(n_4+n_5)]e^{ik_z^{(1)}d_1} + [(n_2+n_3)(n_3+n_4)(n_4+n_5)]e^{-ik_z^{(1)}d_1}}{[(n_2-n_3)(n_3+n_4)(n_4-n_5)]e^{ik_z^{(1)}d_1} + [(n_2+n_3)(n_3-n_4)(n_4-n_5)]e^{-ik_z^{(1)}d_1}} \tag{S10}$$

Where $n_1 = -ik_z^{(2)}d_1 + ik_z^{(2)}d$, $n_2 = \frac{iQ_a}{\varepsilon_a}$, $n_3 = \frac{k_z^{(1)}}{\varepsilon_t^{(1)}}$, $n_4 = \frac{k_z^{(2)}}{\varepsilon_t^{(2)}}$, and $n_5 = \frac{iQ_s}{\varepsilon_s}$.

Therefore, the IFC of polaritons in hBN/MoO_3 can be obtained by solving equation (S10) with equation (S4). In addition, previous works[3,6-10], which are cited in the revised manuscript, demonstrated the derivative process of the IFC of phonon polaritons in MoO_3-based bilayer heterostructure.

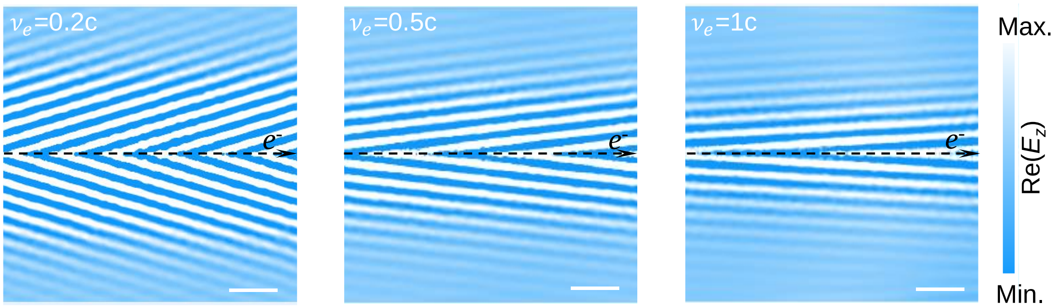

Supplementary Figure S1. The polaritonic reversed CR on MoO_3 excited by different electron velocities. Simulated polaritonic reversed CR of MoO_3 with an excitation frequency of 977 cm^{-1}. The thickness of MoO_3 is 280 nm. Scale bars: 2 μm.

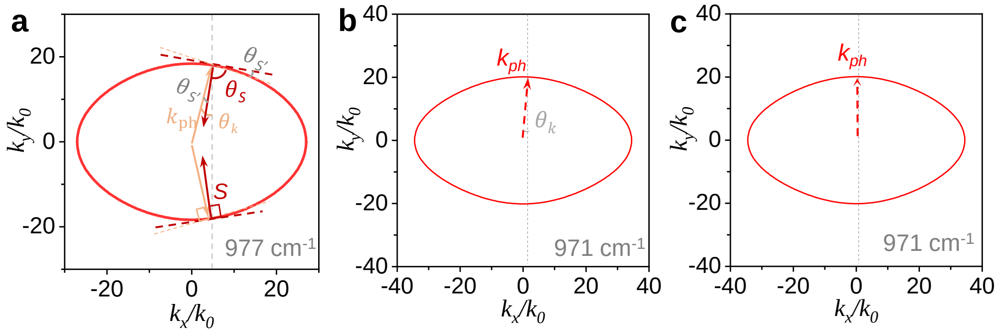

Supplementary Figure S2. Polaritonic reversed CR on MoO_3. a, The calculated elliptical IFC (red line) in the reciprocal space. θ_k is the angle between the wavefront and the moving charged particles, and θ_s is the angle between the wavefront and the radiation energy flow. According to the geometric relationship in reciprocal space, θ_S satisfies: $\theta_S = \pi/2 - \theta_{S'}$, where $\theta_{S'}$ is the angle between the tangent of the IFC and the wavefront. **b,** The calculated elliptical IFC (red line) of Fig. 2b in the reciprocal space. **c,** The calculated elliptical IFC (red line) of Fig. 2c in the reciprocal space. As the incident angle between infrared light and longitudinal axis of the antenna is approaching $\frac{\pi}{2}$, θ_k is becoming smaller.

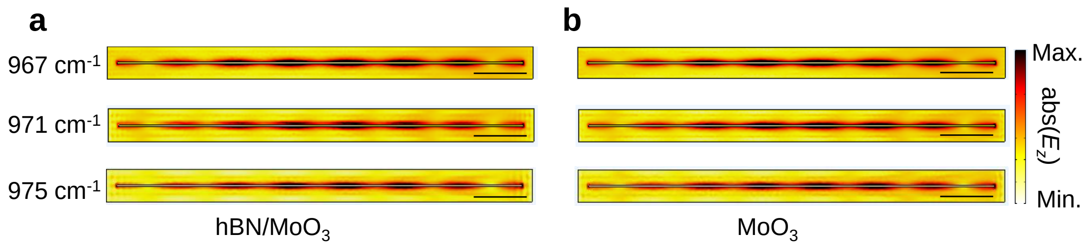

Supplementary Figure S3. The simulated plasmon of metal nanowire on different supported substrates with different excitation frequencies. (a) hBN/MoO_3 substrate. (b) MoO_3 substrate. It is found that the wave vectors of the metal plasmon remain unchanged. The thicknesses of MoO_3 and hBN are 380 nm and 7 nm, respectively. Scale bars: 5 μm.

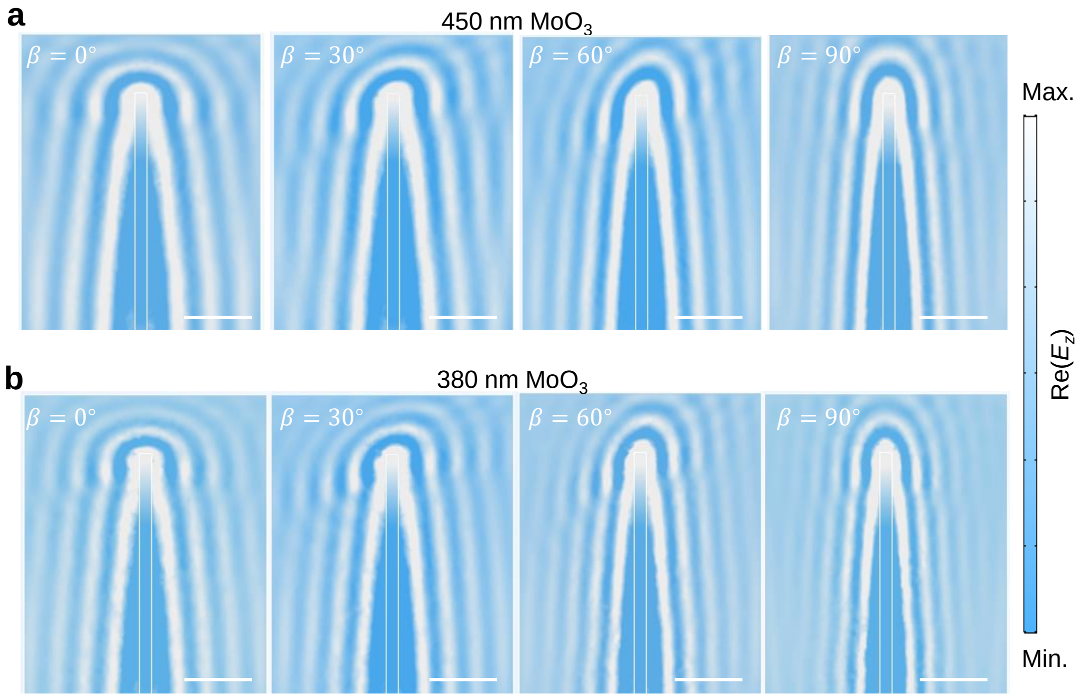

Supplementary Figure S4. Asymmetric polaritonic reversed CR in different thickness MoO_3. Simulated near field distribution images of analogue polaritonic reversed CR on MoO_3 with an excitation frequency of 977 cm^{-1} when β=0°, 30°, 60° and 90°. The thickness of MoO_3 is 450 nm in **a**, and 380 nm in **b,** respectively. Scale bars: 2 μm.

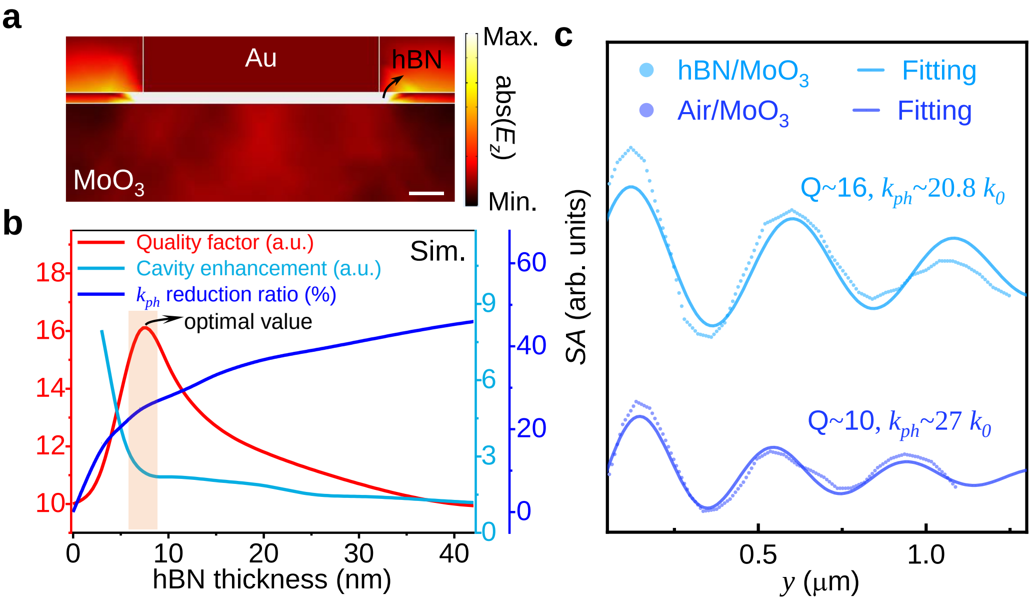

Supplementary Figure S5. Quality factors of hBN/MoO_3 heterostructure and MoO_3. **a,** Electromagnetic field intensity (abs (E_z)) distribution of Au antenna/hBN/MoO_3 in the *y-z* section. The ultra-thin hBN film is constructed into nanocavity to realize the large electromagnetic field enhancement. Scale bar: 50 nm. **b,** Summarized quality (Q) factor of polaritonic CR on MoO_3 under different thicknesses of hBN (details are shown Fig. S6&7), which is mainly affected by the cavity field enhancement and wave vector (k_{ph}) reduction ratio in the hBN/MoO_3 heterostructure. It is noticed that the cavity enhancement factor is larger, but the k_{ph} reduction ratio is smaller when the thickness of hBN is decreasing. Thus, these two factors compete with each other and result in first increased and then decreased excitation efficiency of analogue polaritonic reversed CR. When the excitation efficiency of analogue polaritonic reversed CR is higher for the hBN/MoO_3 heterostructure, the Q factor is higher. **c,** The extracted interference fringes of phonon polaritons in MoO_3 and hBN/MoO_3 (Fig. 4c) with an excitation frequency of 977 cm^{-1}. Experiment data: points. Fitting data: lines.

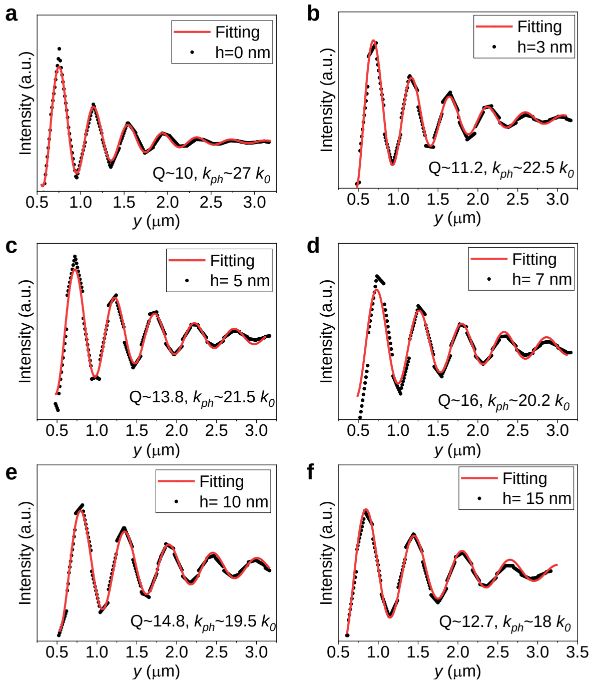

Supplementary Figure S6. Simulated performance of hBN/MoO_3 heterostructure when varying the thickness of hBN. a-f, The thickness of hBN layer is from 0 nm to 15 nm.

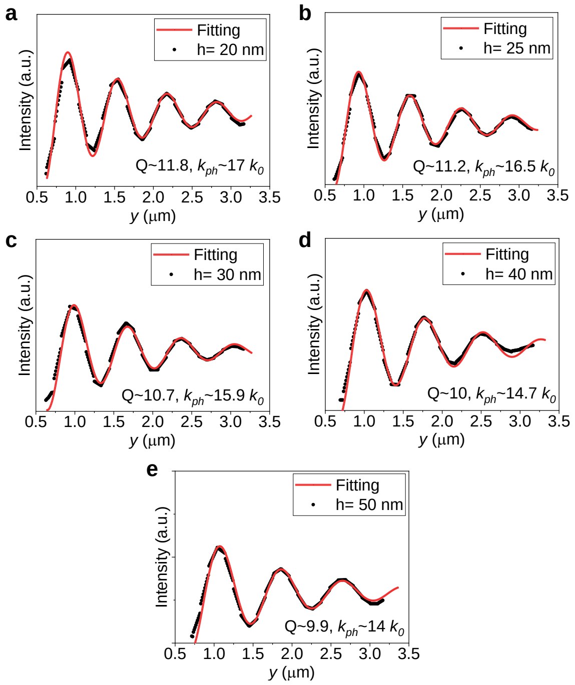

Supplementary Figure S7. Simulated performance of hBN/MoO_3 heterostructure when varying the thickness of hBN. a-e, The thickness of hBN layer is from 20 nm to 50 nm.

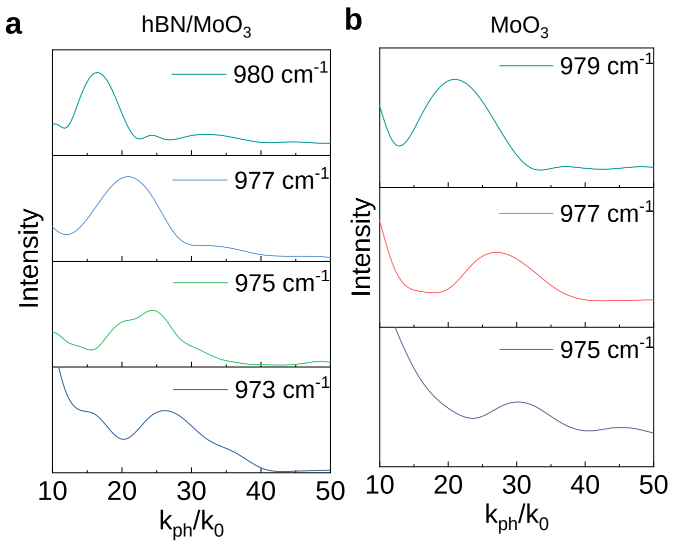

Supplementary Figure S8. Polaritonic reversed CR angle of hBN/MoO_3 heterostructure and MoO_3. a, The fast Fourier transform (FFT) of the analogue polaritonic reversed CR pattern on hBN/MoO_3. **b,** The FFT of the analogue polaritonic reversed CR pattern on MoO_3. Then the k_{ph} is obtained to calculate θ_k, as shown in Fig. 4d.

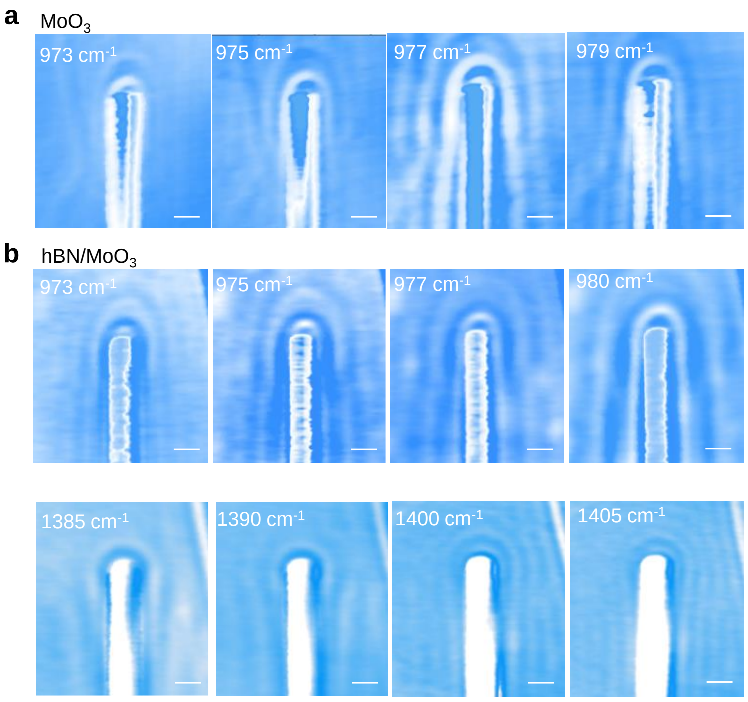

Supplementary Figure S9. Polaritonic CR in MoO_3 and hBN/MoO_3 heterostructure. a, Experimental data of analogue polaritonic reversed CR on the

MoO_3 with varied excitation frequencies. **b,** Forward and reversed CR on the hBN/MoO_3 heterostructure with varied excitation frequencies, respectively. The thickness of MoO_3 is approximately 280 nm, and the thickness of hBN is approximately 7 nm. Scale bars: 500 nm.

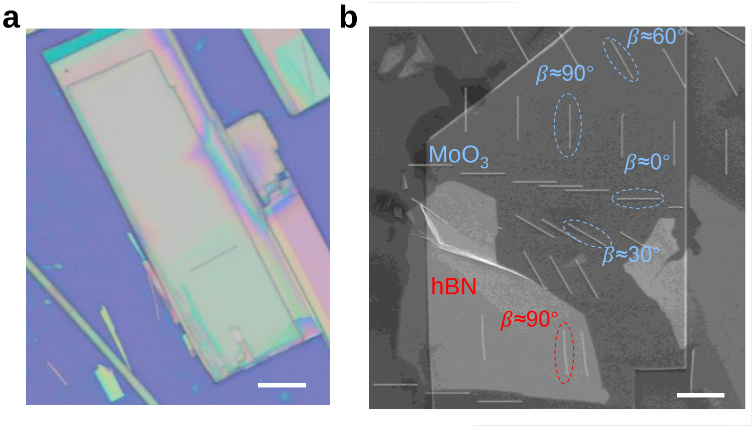

Supplementary Figure S10. Metal nanowires on MoO_3 flakes and hBN/MoO_3 heterostructure. a, Optical image of silver nanowires on the MoO_3 flake, where analogue polaritonic reversed CR is studied in Fig. 2. **b,** Scanning electron microscopy image of gold nanowires on MoO_3 flake and hBN/MoO_3 heterostructure, where the symmetric reversed CR and the asymmetric reversed CR of plasmons on MoO_3 are studied in Fig. 3, and reversed CR in hBN/MoO_3 heterostructure is investigated in Fig. 4. Scale bars: 10 μm.

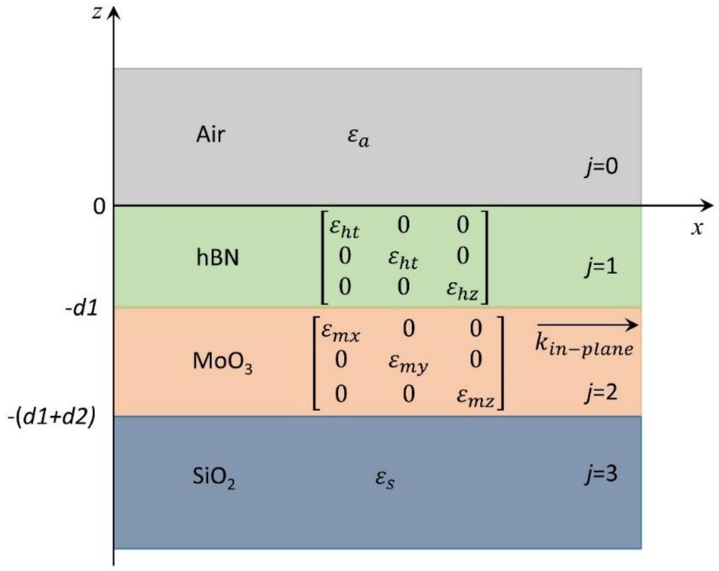

Supplementary Figure S11. Schematic illustration of hBN/MoO_3 heterostructure which is treated as a laterally infinitely layered medium consisting of four layers.

Supplementary References

1. Li, N. *et al.* Direct observation of highly confined phonon polaritons in suspended monolayer hexagonal boron nitride. *Nat. Mater.* **20**, 43-48, (2021).
2. Raza, S. *et al.* Extremely confined gap surface-plasmon modes excited by electrons. *Nat. Commun.* **5**, (2014).
3. Alvarez-Perez, G. *et al.* Infrared Permittivity of the Biaxial van der Waals Semiconductor alpha-MoO3 from Near- and Far-Field Correlative Studies. *Adv. Mater*. **32**, e1908176, (2020).
4. Zheng, Z. *et al.* A mid-infrared biaxial hyperbolic van der Waals crystal. *Sci. Adv*. **5**, eaav8690, (2019).
5. Ma, W. *et al.* In-plane anisotropic and ultra-low-loss polaritons in a natural van der Waals crystal. *Nature* **562**, 557-562, (2018).
6. Chen, M. *et al.* Configurable phonon polaritons in twisted alpha-MoO(3). *Nat. Mater.* **19**, 1307-1311, (2020).
7. Sun, F. *et al.* Polariton waveguide modes in two-dimensional van der Waals crystals: an analytical model and correlative nano-imaging. *Nanoscale* **13**, 4845-4854, (2021).
8. Hu, G. *et al.* Topological polaritons and photonic magic angles in twisted alpha-MoO(3) bilayers. *Nature* **582**, 209-213, (2020).
9. Duan, J. *et al.* Twisted Nano-Optics: Manipulating Light at the Nanoscale with Twisted Phonon Polaritonic Slabs. *Nano Lett.* **20**, 5323-5329, (2020).
10. Zheng, Z. *et al.* Phonon Polaritons in Twisted Double-Layers of Hyperbolic van der Waals Crystals. *Nano Lett.* **20**, 5301-5308, (2020).
